# Supplementary material for: A single valine to leucine switch disrupts Plasmodium falciparum AP2-G DNA binding and reveals GDV1’s role in ap2-g activation
Source: bioRxiv. 2025 Apr 23:2025.04.22.648970. Preprint. [Version 1] doi: 10.1101/2025.04.22.648970 (PMC12190761; doi:10.1101/2025.04.22.648970)
Supplement: Supplement 1 [file media-1.pdf]

A single valine to leucine switch disrupts *Plasmodium falciparum* AP2-G DNA binding and reveals GDV1's role in *ap2-g* activation.

Surendra K. Prajapati[1,2*], Jeffrey X. Dong[1,2], Belinda J. Morahan[3], Thoai Dotrang[1,2], Michelle C. Barbeau[1,2], April E. Williams[4@], Daniel Hupalo[5], Matthew D. Wilkerson[5], Clifton L. Dalgard[5], Bjorn F.C. Kafsack[6], Manuel Llinás[7, 8], Kim C. Williamson[1#,3]

[1]Department of Microbiology and Immunology, Uniformed Services University of the Health Sciences, Bethesda, USA, [2]Henry M. Jackson Foundation for the Advancement of Military Medicine, Inc., Bethesda, USA, [3]Laboratory of Malaria and Vector Research, NIAID, NIH, Rockville, USA, [4]Department of Molecular Biology, Lewis-Sigler Institute for Integrative Genomics, Princeton University, Princeton, USA, [5]Department of Anatomy, Physiology & Genetics, Uniformed Services University of the Health Sciences, Bethesda, USA, [6]Department of Microbiology & Immunology, Weill Cornell Medicine, New York, USA, [7]Department of Biochemistry & Molecular Biology and the Huck Center for Malaria Research, Pennsylvania State University, University Park, USA, [8]Department of Chemistry, Pennsylvania State University, University Park, USA. *Corresponding author (surendra.prajapati.ctr@usuhs.edu), [@]present address: Integrative Genomics and Bioinformatics Core, The Salk Institute for Biological Studies, La Jolla, CA, USA, [#]current affiliation.

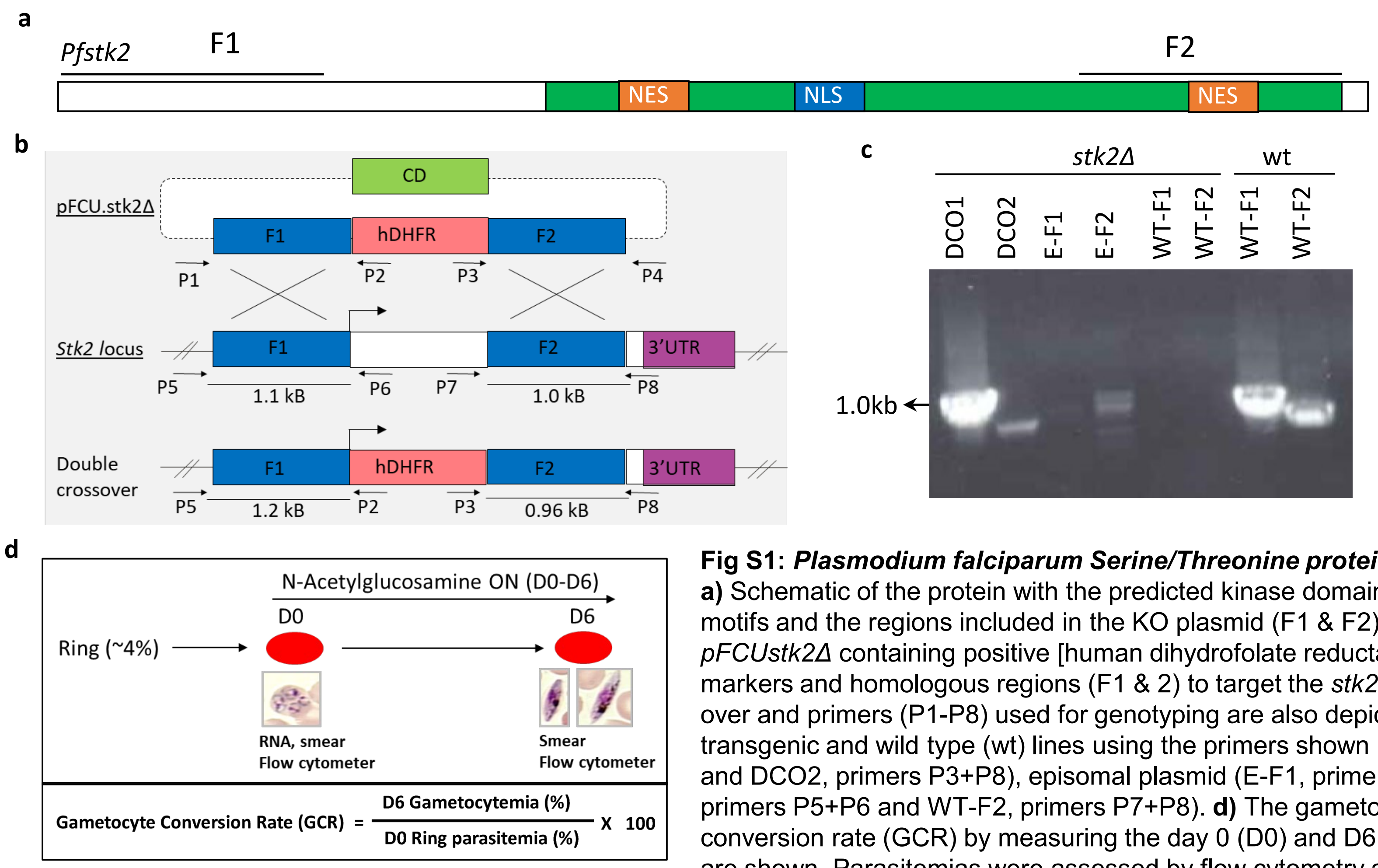

Fig S1: ***Plasmodium falciparum Serine/Threonine protein kinase*** **(***Pfstk2***)** knockout strategy and gametocyte phenotyping assay. **a)** Schematic of the protein with the predicted kinase domain (green), nuclear export signal (NES), nuclear localization signal (NLS) motifs and the regions included in the KO plasmid (F1 & F2) indicated**. B)** The double crossover *knockout* strategy using plasmid *pFCUstk2Δ* containing positive [human dihydrofolate reductase (DHFR)] and negative [Cytosine deaminase (CD)] selectable markers and homologous regions (F1 & 2) to target the *stk2* is shown. The *Pfstk2* locus before and after a successful double cross over and primers (P1-P8) used for genotyping are also depicted. **c)** Polymerase chain reaction (PCR) genotyping of the *3D7.stk2Δ* transgenic and wild type (wt) lines using the primers shown in **b** to detect double crossover (DCO) integration (DCO1, primers P2+P5 and DCO2, primers P3+P8), episomal plasmid (E-F1, primers P1+P2 and E-F2, primers P3+P4) and the wild type locus (WT-F1, primers P5+P6 and WT-F2, primers P7+P8). **d)** The gametocyte phenotyping assay and formula used to determine the gametocyte conversion rate (GCR) by measuring the day 0 (D0) and D6 parasitemias of cultures grown in the presence of N-acetylglucosamine are shown. Parasitemias were assessed by flow cytometry and Giemsa-stained culture smears.

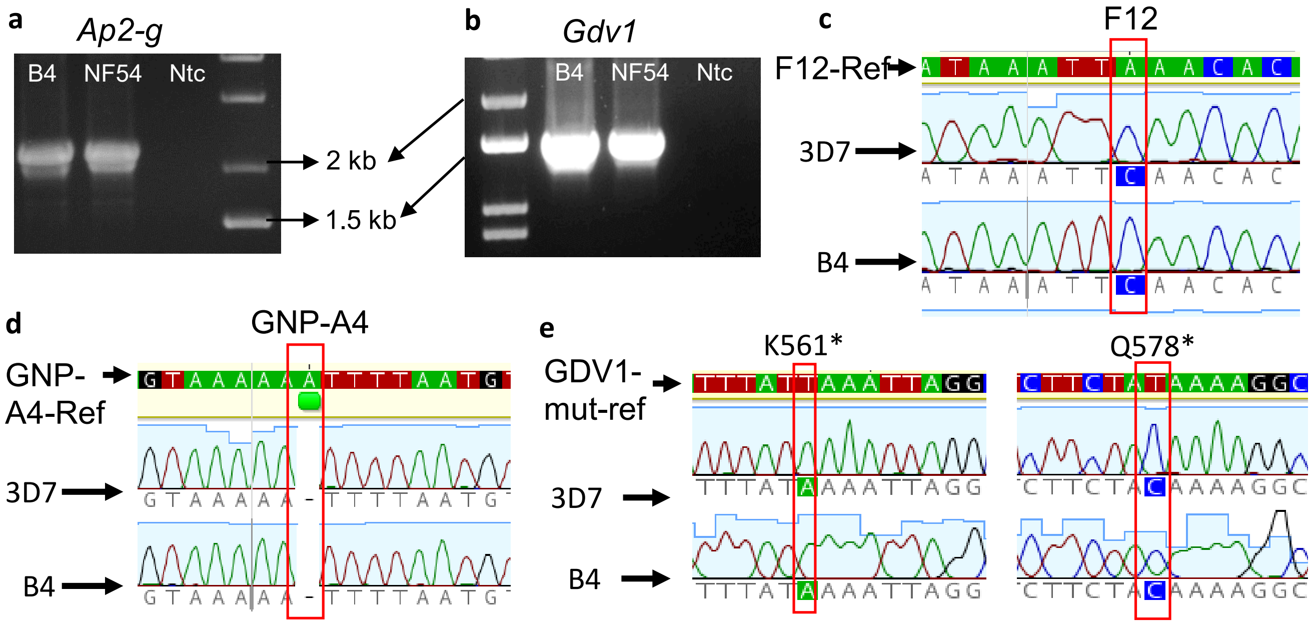

Fig S2: *Ap2-g* and *gdv1* loci test for known mutations conferring premature termination of protein. *Ap2-g* from coding region (bp 6024) to 3'-UTR (bp 988) **(a)** and *gdv1* from coding region (bp 841) to 3'-UTR (bp 532) **(b)** were amplified using polymerase chain reaction from *3D7.stk2Δ* clone B4, or wild type (wt) parasite DNA and a no template control (Ntc). **c-e)** Chromatograms of the DNA sequence of wt 3D7 or *3D7.stk2Δ clone B4* (B4) parasites are shown below the reference sequence for the known *ap2-g* [1] **(c,d)** or *gdv1* [2] **(e)** [3] **(f)** mutations that block gametocyte production. Red boxes (**c-e**) indicates the nucleotide positions where mutations in *ap2-g* were expected for parasite lines F12 and GNP-A4 and in *gdv1* (K561*, Q578*).

<u>Reference:</u>

1) Kafsack BF, et al. A transcriptional switch underlies commitment to sexual development in malaria parasites. Nature 507, 248-252 (2014).
2) Tiburcio M, et al. A 39-Amino-Acid C-Terminal Truncation of GDV1 Disrupts Sexual Commitment in Plasmodium falciparum. mSphere 6, (2021).
3) Llora-Batlle O, et al. Conditional expression of PfAP2-G for controlled massive sexual conversion in Plasmodium falciparum. Sci Adv 6, eaaz5057 (2020).

2163th	AP2-G (2159-2218)	Pridicted AP2 domain Position	i-Evalue
Wt (V)	PIHSVWKDTTRGHCSWRCRWWENGRRLSKNFNVKRFGNDGALRMAITMKLKKSNPKEQMQ	2162 - 2210	0.00053
V->M	PIHSMWKDTTRGHCSWRCRWWENGRRLSKNFNVKRFGNDGALRMAITMKLKKSNPKEQMQ	2173 - 2210	0.0023
V->V	PIHSFWKDTTRGHCSWRCRWWENGRRLSKNFNVKRFGNDGALRMAITMKLKKSNPKEQMQ	2173 - 2210	0.0023
V->W	PIHSWWKDTTRGHCSWRCRWWENGRRLSKNFNVKRFGNDGALRMAITMKLKKSNPKEQMQ	2173 - 2210	0.0023
V->Y	PIHSYWKDTTRGHCSWRCRWWENGRRLSKNFNVKRFGNDGALRMAITMKLKKSNPKEQMQ	2173 - 2210	0.0023
V->S	PIHSSWKDTTRGHCSWRCRWWENGRRLSKNFNVKRFGNDGALRMAITMKLKKSNPKEQMQ	2173 - 2210	0.0023
V->T	PIHSTWKDTTRGHCSWRCRWWENGRRLSKNFNVKRFGNDGALRMAITMKLKKSNPKEQMQ	2173 - 2210	0.0023
V->N	PIHSNWKDTTRGHCSWRCRWWENGRRLSKNFNVKRFGNDGALRMAITMKLKKSNPKEQMQ	2173 - 2210	0.0023
V->Q	PIHSQWKDTTRGHCSWRCRWWENGRRLSKNFNVKRFGNDGALRMAITMKLKKSNPKEQMQ	2173 - 2210	0.0023
V->C	PIHSCWKDTTRGHCSWRCRWWENGRRLSKNFNVKRFGNDGALRMAITMKLKKSNPKEQMQ	2173 - 2210	0.0023
V->P	PIHSPWKDTTRGHCSWRCRWWENGRRLSKNFNVKRFGNDGALRMAITMKLKKSNPKEQMQ	2173 - 2210	0.0023
V->R	PIHSRWKDTTRGHCSWRCRWWENGRRLSKNFNVKRFGNDGALRMAITMKLKKSNPKEQMQ	2173 - 2210	0.0023
V->H	PIHSHWKDTTRGHCSWRCRWWENGRRLSKNFNVKRFGNDGALRMAITMKLKKSNPKEQMQ	2173 - 2210	0.0023
V->K	PIHSKWKDTTRGHCSWRCRWWENGRRLSKNFNVKRFGNDGALRMAITMKLKKSNPKEQMQ	2173 - 2210	0.0023
V->D	PIHSDWKDTTRGHCSWRCRWWENGRRLSKNFNVKRFGNDGALRMAITMKLKKSNPKEQMQ	2173 - 2210	0.0023
V->E	PIHSEWKDTTRGHCSWRCRWWENGRRLSKNFNVKRFGNDGALRMAITMKLKKSNPKEQMQ	2173 - 2210	0.0023

Fig S3: *In-silico* replacement of valine with other amino acids in AP2-G AP2 domain predictions using MOTIF search software. AP2-G amino acids (aa) 2159-2218 are shown from wild type 3D7 and with valine$_{2163}$ substituted with the indicated amino acid. Red text indicates the AP2 domain predicted for each sequence using MOTIF search software and the green box indicates position 2163. The last two columns are the aa included in the predicted AP2 domain and the i-Evalue of the sequence that predicts the DNA interaction strength.

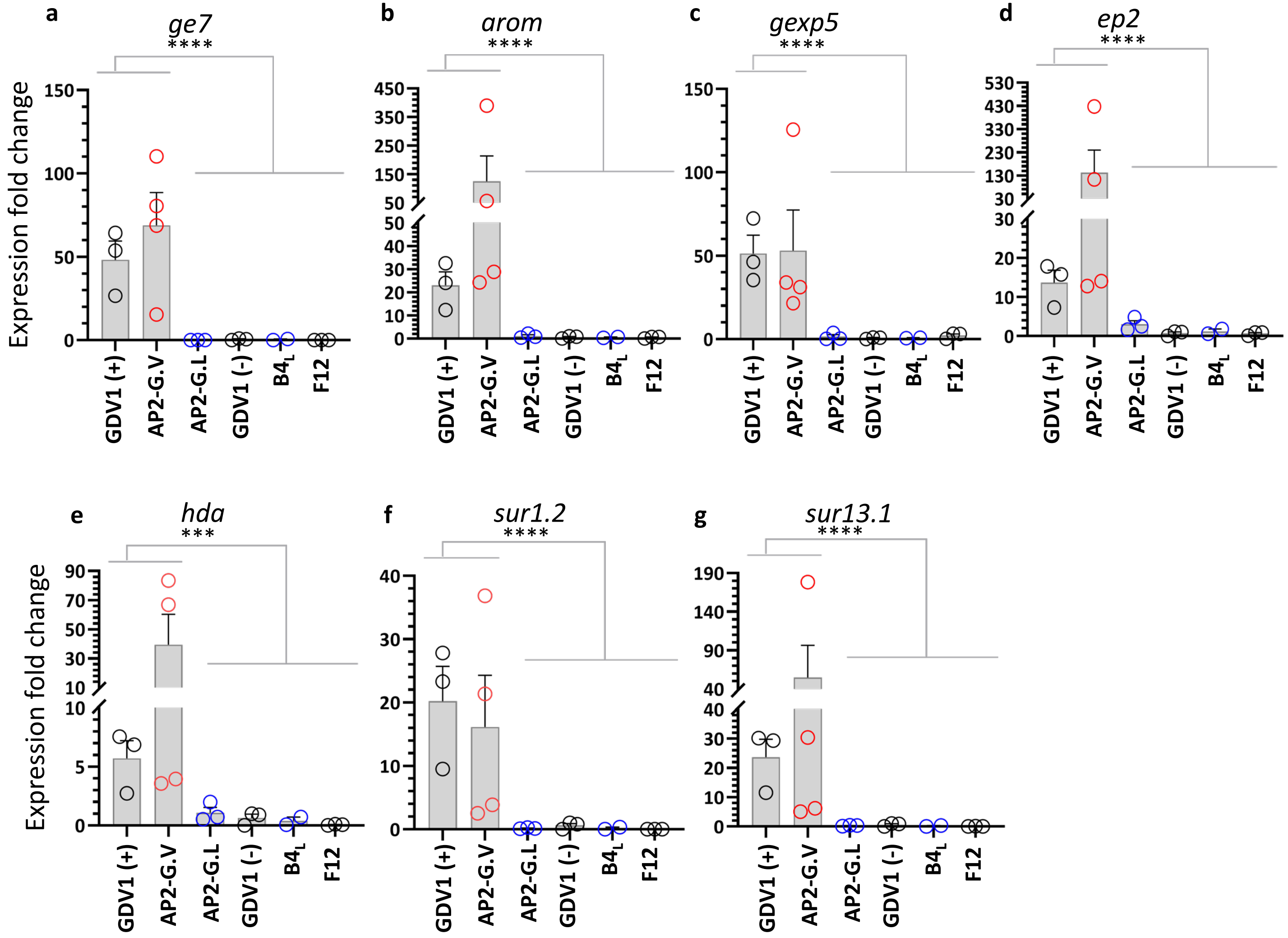

Fig S4: Ring stage expression of AP2-G dependent genes. AP2-G dependent gene expression was analyzed using reverse transcriptase–quantitative polymerase chain reaction (RT-qPCR) of RNA isolated from sorbitol synchronized ring stage parasites (6-10 hpi) encoding *ap2-g.V [NF54.gdv1.3xha.fkbp* with (GDV1(+) or without (GDV1(-)) Shld1, *3D7.stk2Δ//ap2-g.V* (AP2-G-V)], *ap2-g. L*, [*NF54.ap2-g.L* (AP2-G.L), *3D7.stk2Δ//ap2-g.L* ($B4_L$)] or truncated *ap2-G* (F12). The GDV1 (-) parasite line was used as the comparator to determine fold change. The RT-qPCR primers used are listed in **Supplemental Table 2** with the PlasmoDB ID # of the gene. Each circle indicates an independent data point and the bar and error bar represent the mean and standard error of the mean, respectively. A Kruskal-Wallis test followed by Dunn's multiple comparison was used for statistical analysis between groups. The p value of each comparison is indicated by *** (<0.001) and **** (<0.0001).

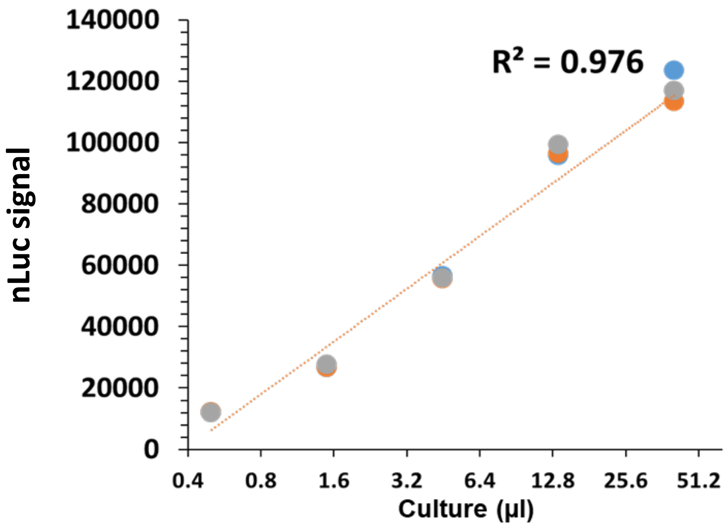

Fig S5. Nanoluciferase (nLuc) signal is positively correlated with parasite number (culture volume) using an *ap2-g* reporter line, *NF54.ap2-g.V.p2a.nLuc*. Colored circles indicate biological replicates. All circles are shown for the culture volumes, but for each of the first 3 volumes the points overlap.

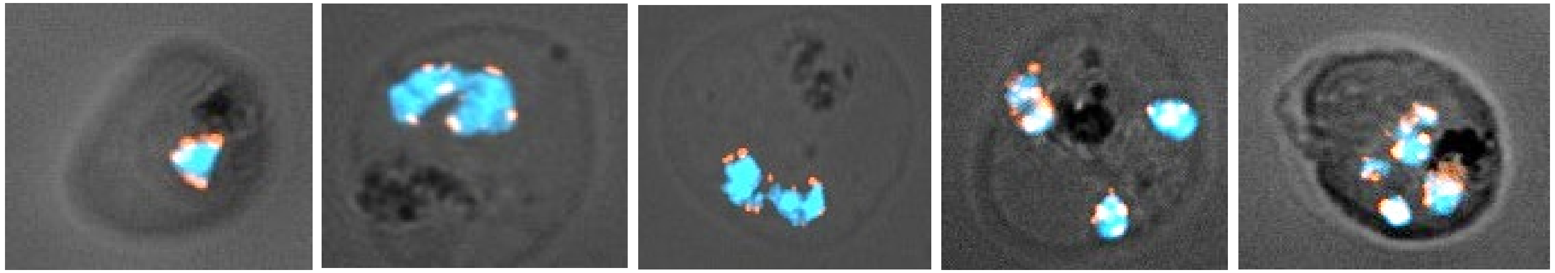

Fig S6. GDV1 expression during schizogony begins in late trophozoites and continues into early schizonts. *683.Gdv1.fkbp.tdTom* transgenic parasites were stained with Hoechst (1:500 dilution) and imaged using fluorescence microscopy. An overlay of the bright field, Hoechst and tdTomato images are shown.

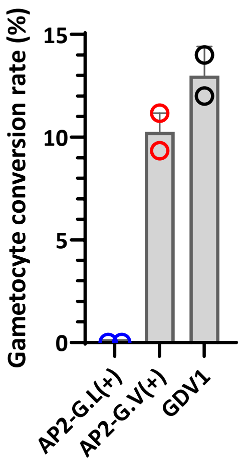

Fig. S7: Day 6 gametocyte conversion rate of the parasite lines used in the nanoluciferase (nLuc) time course (Figure 5a). Day 6 gametocyte conversion rates of AP2-G.L(+), *3D7.stk2Δ//ap2-g.L.fkbp.p2a.nLuc* (Shld1+,blue circle), AP2-G.V (+), *3D7.stk2Δ //ap2g.V.fkbp.p2a.nLuc* (Shld1+,red circle), and GDV1, *NF54.gdv1.p2a.nLuc* (black circle) are shown. Each circle indicates an independent data point and the bar and error bars represent mean and standard error of the mean, respectively.

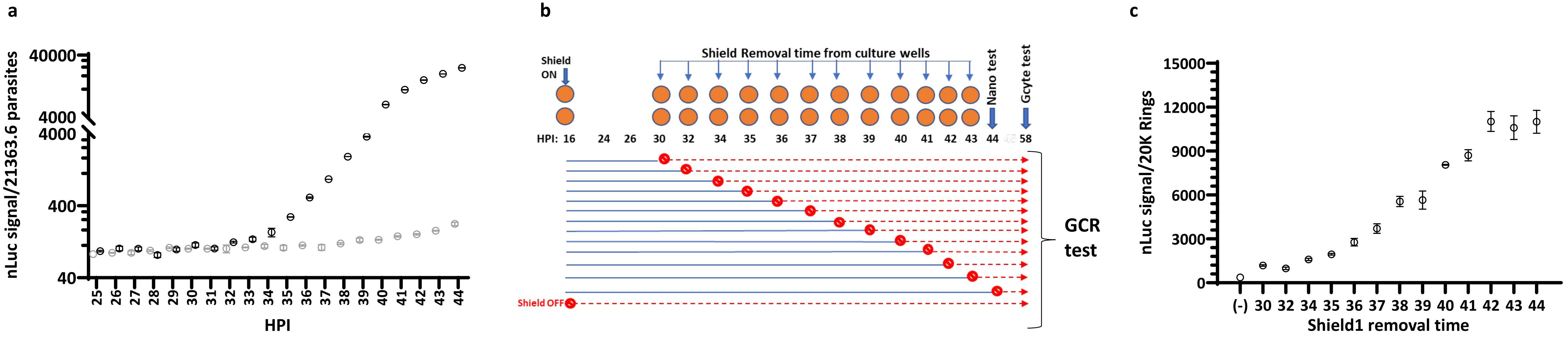

Fig. S8: Time-course to determine when GDV1 is required for maximal *ap2-g* expression. a) Time-course of *ap2-g*-linked nanoluciferase (nLuc) activity using the *NF54.gdv1.3xha.fkbp//ap2-g.p2 nLuc* line in the presence (black) and absence (gray) of Shld1. **b)** Schematic showing the time points when GDV1 was first added and then removed from the culture wells. Blue horizontal lines indicate culture wells with Shld1 whereas red dotted lines indicate no Shld1. At 58 hpi, after monitoring nLuc, N-acetylglucosamine (NAG) was added to each well and this was designated Day 0 for gametocyte assessment and gametocytemia was measured 7 days later (D6) to calculate gametocyte conversion rate (GCR). **c)** The 56 hpi *ap2-g-* linked nLuc signal of each well before adding NAG. Each circle indicates an independent data point and the error bars represent the standard error of the mean, respectively.

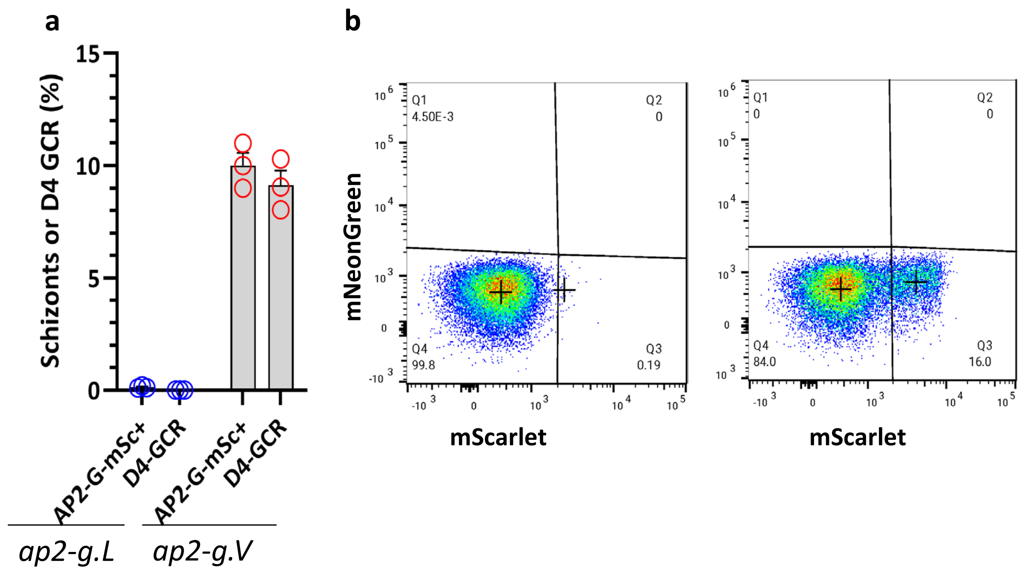

Fig. S9: Flow cytometric analysis of AP2.V and AP2.L reporter lines.
a) Comparison of the % of AP2-G.mSc+ schizonts detected by flow cytometry and the day 4 gametocyte conversion rate (D4-GCR) of *ap2-g.L* (*3D7.stk2Δ//ap2-g.L.mSc*) and *ap2-g.V* (*3D7.stk2Δ//ap2-g.V.mSc*) mScarlet reporter lines. **b)** Flow cytometric analysis of *ap2-g.L* (*3D7.stk2Δ//ap2-g.L.mSc*) (left panel) and *ap2-g.V* (*3D7.stk2Δ//ap2-g.V.mSc*) (right panel) mScarlet reporter lines.

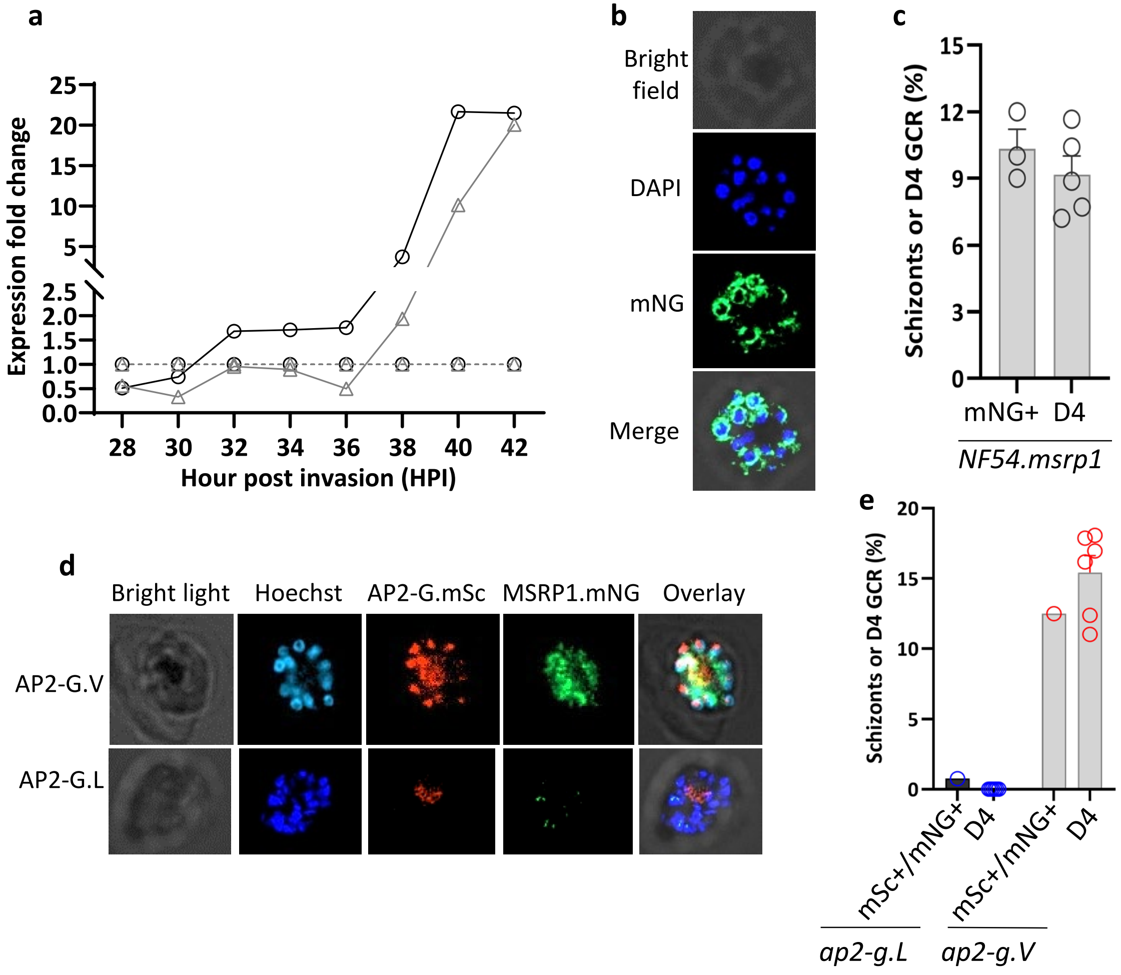

Fig S10: *Msrp1* and *ap2-g* expression time-course during schizogony
a) The increase in *msrp1* (triangles) and *ap2-g* (circles) RNA over time in a *gdv1* inducible line *NF54.gdv1.3xha.fkbp* in the presence of Shld1 (continuous line) or absence of Shld1 (dotted line). **b**) Live imaging of the *NF54.msrp1.mNG* line stained with Hoechst to detect DNA demonstrates MSRP1.mNG expression in mature schizonts. **c)** Comparison of the % of MSRP1.mNG positive schizonts measured by flow cytometry and the day 4 gametocyte conversion rate in *NF54.msrp1.mNG* parasites. **d)** Live imaging of AP2-G.mSc and MSRP1.mNG expression in the *NF54.msrp1.mNG//ap2-g.V.mSc* (top panel) and *NF54.msrp1.mNG//ap2-g.L.mSc* (bottom panel) lines stained with Hoechst to detect DNA. **e)** Comparison of the % of double positive schizonts (AP2-G.mSc+/MSRP1.mNG+) and the day 4 gametocyte conversion rate in *ap2-L*, *3D7.stk2Δ//ap2-g.L.mSc//msrp1.mNG*) or *ap2-g.V* (*3D7.stk2Δ//ap2-g.V.mSc//msrp1.mNG*) expressing lines. Blue and red circles indicate *ap2-g.L* and *ap2-g.V* lines, respectively. Each circle indicates independent data points and the bar and error bars represent the mean and standard error of the mean, respectively.

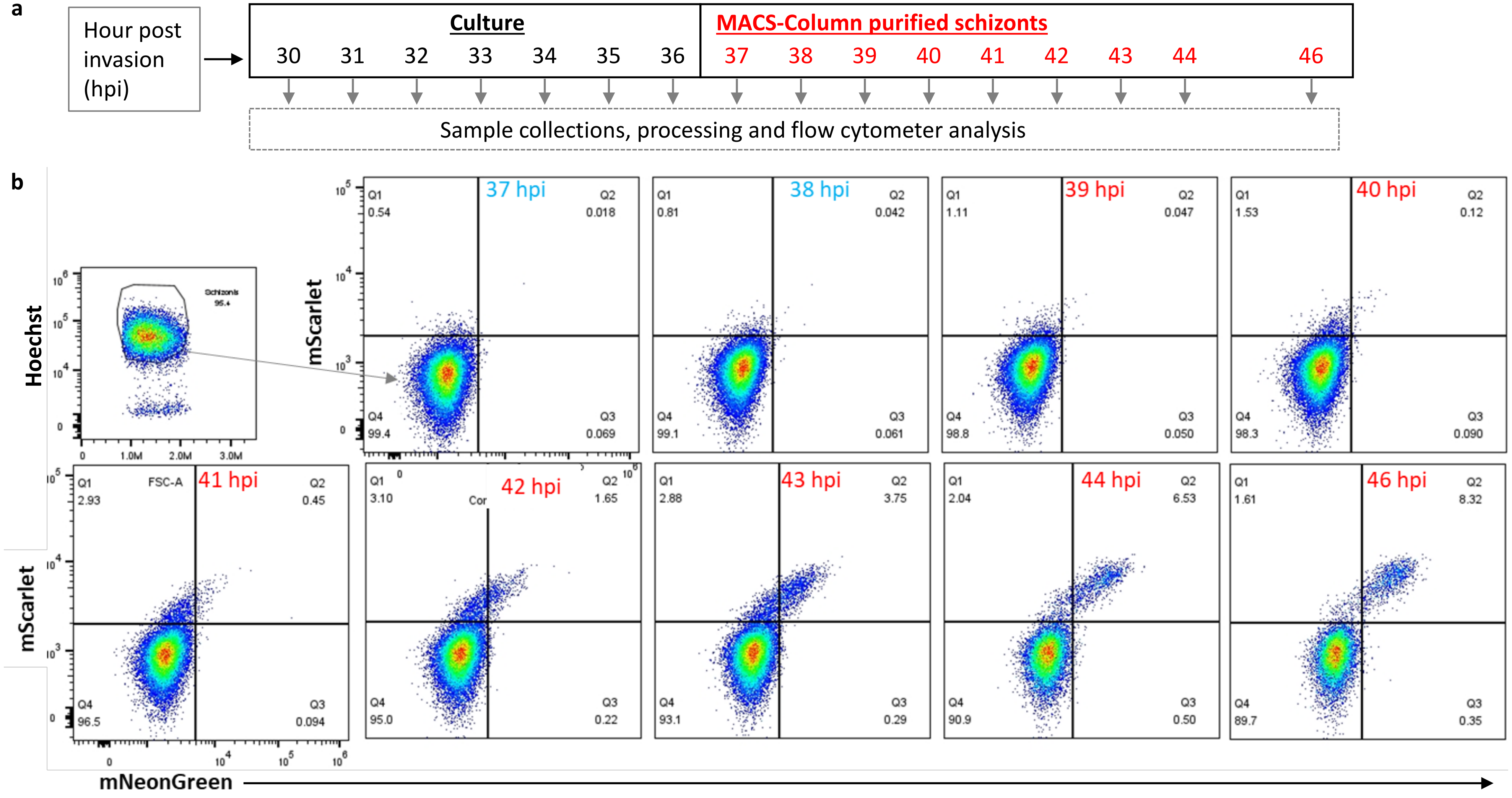

Fig. S11: Time-course of AP2-G.mScarlet and MSRP1.mNeonGreen expression through schizogony. a) Schematic of the experimental design. Beginning 30 h post RBC invasion (hpi) samples were taken from a synchronized culture for flow cytometric analysis. At 36 hpi schizonts had matured enough to allow MACS-column purification before flow cytometry, which reduced the number of uninfected RBCs. **b**) Quantification of AP2-G.mSc and MSRP1.mNG positive schizonts using flow cytometry. None of the early schizonts were positive for AP2-G.mSc before 37 hpi and therefore not included in the figure. AP2-G.mSc positive schizonts gradually increased from 38 hpi reaching 1% of the schizont population at 39 hpi. The % of AP2-G.mSc single positive schizonts plateaued at ~3% at 41 hr as MSRP1.mNG expression was detected and the number of double positive schizonts began to increase. After 43 hr the number of AP2-G single positive schizonts declined rapidly, while the number of double positive schizonts continued to increase, again consistent with AP2-G expression being required for MSRP1 expression.

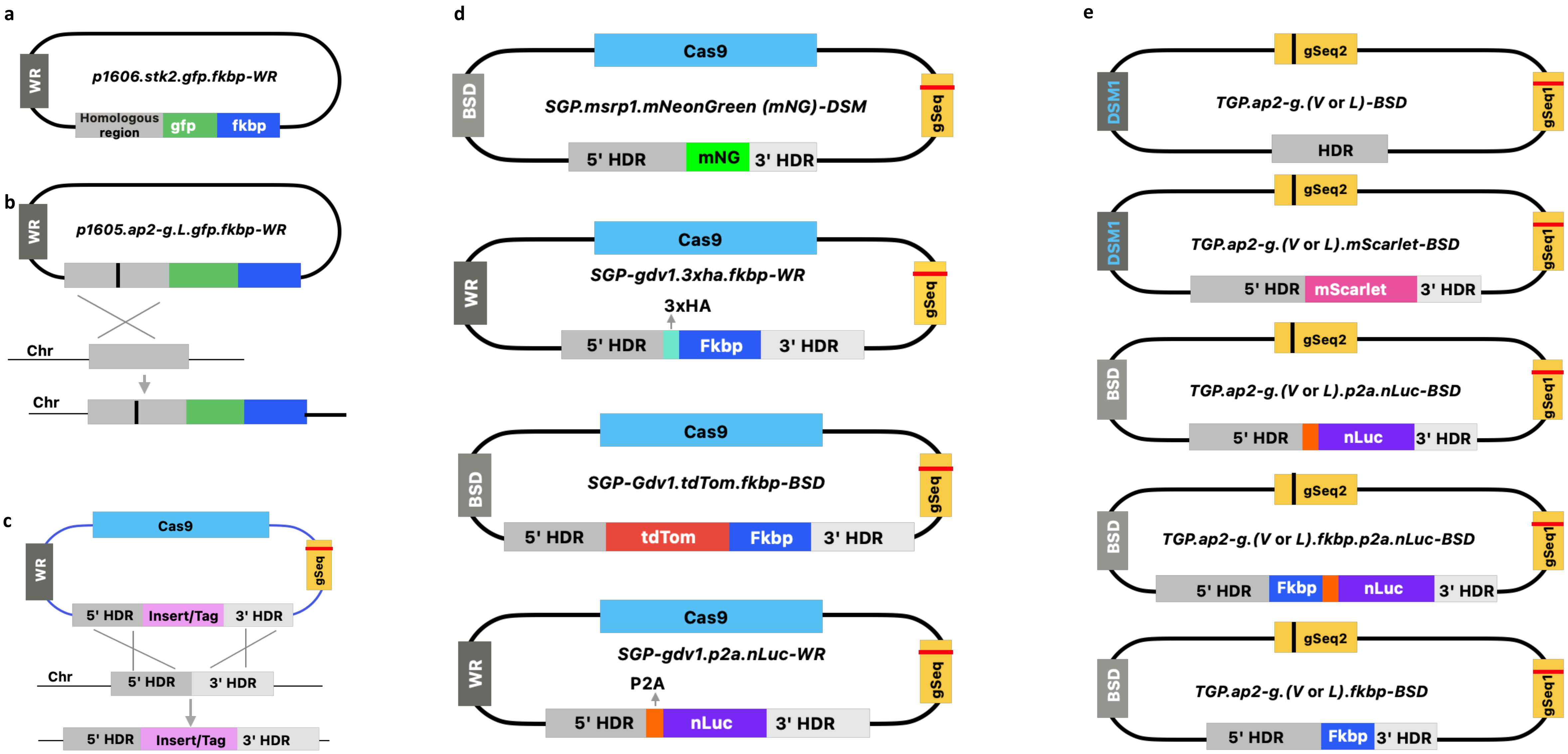

Fig. S12: Plasmids used to tag/modify *stk2, gdv1, ap2-g* and *msrp1*. The selectable markers were *dihydrofolate reductase* (WR drug), *blasticidin S deaminase* (BSD drug), *dihydroorotate dehydrogenase* (DSM1 drug). Black line in the homologous region **(b)** indicates *ap2-g.L* mutation. HDR and gSeq **(d-e)** indicated homology directed repair template and guide RNA sequence, respectively. Fkbp is an inducible expression system that targets the attached protein for degradation in the absence of Shld1 ligand.

Table S1: Whole genome sequences showing mutations in B4 clone (*3D7.stk2Δ//ap2-g.L*) and wild type NF54.

Chromosome	Position	Annotation	Gene	Ref Allele	Alt allele	GT:AD:DP (B4 clone)	GT:AD:DP (NF54)
Pf3D7_10_v3	1187187	missense	PF3D7_1029000	G	A	1/1:2,27:30	1/1:4,26:34
Pf3D7_10_v3	1437030	missense	PF3D7_1036400	A	G	1/1:0,8:8	./. (Deletion)
Pf3D7_12_v3	913689	missense	PF3D7_1222600	**G**	**T**	1/1:0,101:101	0/0:.:143
Pf3D7_12_v3	1908160	missense	PF3D7_1245800	A	T	1/1:0,17:17	1/1:0,20:20
Pf3D7_12_v3	1908195	missense	PF3D7_1245800	T	A	1/1:0,32:32	1/1:0,28:28

GT: Genotype, AD: Read depth for each allele, DP: Read depth

Table S2: List of primers used for plasmid construction, transgenic line genotyping and genes expression analysis.

Oligo types	Oligo name	Oligo sequence (5' - 3') (Ref)	Ref
**Plasmid construction**			
Stk2-KO (PF3D7_0214600)	*P21 (P1)*	*TCCCCGCGGTTAAGTACAATTACATGTAATTAA*	
	P22 (P2)	*CTAGACTAGTGTTCTTATGATTAGTAATAATTTC*	
	P68 (P3)	*CATGCCATGGTTGAAGAGCCCAAAATGTTAT*	
	P69 (P4)	*GATCCTAGGCAAATCTCTAAAAGAATAATG*	
Stk2 (PF3D7_0214600)	*STK2(Xho1)_F*	*TAATCTCGAGTATTCAATTGAAGAGCCC*	
	STK2(Xho1)_R	*TAATCCTAGGTATCAAATCTCTAAAAGAATAATG*	
Gdv1 (PF3D7_0935400)	GDV1_recoGseq1_F	tattGGATTTATTCTCATAACAAC	
	GDV1_recoGseq1_R	aaacGTTGTTATGAGAATAAATCC	
	GDV1_InFu_F	GTACCGAGCTCGAATTCTTGATAAAATATGGGAGATC	
	GDV1_recoHDR1_R	GGGTTGATACGCATTACGACTGGAATACCTTTCTGTTTTA	
	GDV1_recoHDR1_F	AGTCGTAATGCGTATCAACCCTTTTCAATCTTTTCGTTTA	
	GDV1_InFu_Rn	CATATGGGTAGCGGCCGCTTTTATATGTACATTTTTC	
	GDV1 3UTR_InFu_F	AACTAGTGTGACCGGTTAAATAAATGAAAAAAAAAA	
	GDV1 3UTR_InFu_R	AGTGCCACCTGACGTCATGTTTTTATTTGTGCTTA	
	GDV1_InFu_tdTom_F	CATATAAAAGCGGCCGCATGGTGAGCAAGGGCGAGG	
	GDV1_InFu_FKBP_R	CATTTATTTAACCGGTTTCTTCCGGTTTTAGAAGC	
	Gdv1_P2ANano_F	ACATATAAAAGCGGCCGCGCCACAAACTTCTCTCTGCT	
	Gdv1_Nano_R	TTAACCGGTCACACTAGTCGCCAGAATGCGTTCGCACAG	
Ap2-g (PF3D7_1222600)	*AP2-g Val_1 (Xho1) F*	*taactcgagAGGATGAAGATGATGATAATAAC*	
	AP2-g Val (AvrII) R	*taacctaggAATATTCCTGTTGTTTCCCCCTT*	
	Ap2G-CRISP_F	tattTTGCCACAAAAAGGAATAAG	
	Ap2G-CRISP_R	aaacCTTATTCCTTTTTGTGGCAA	
	Ap2g_3UTRgSeq_F	tattGACATACAGTTCTTATAATG	
	Ap2g_3UTRgSeq_R	aaacCATTATAAGAACTGTATGTC	
	InFu_AP2g_FKBP_F	CAGGAATATTAGCGGCCGCGGAGTGCAGGTGGAAACCAT	
	InFu_AP2g_FKBP_R	GGATGATATTTAACGCGTTTCTTCCGGTTTTAGAAGC	
	Ap2g_3UTR_PAM_F	ATATTTTTTTTTATACATTATAAGAACTG	
	InFu_Ap2g_3UTR_R	CGAAAAGTGCCACCTGACGTCTATGAAATGTTACAGTTTG	
	InFu_AP2g_mScarlet_F	CAGGAATATTAGCGGCCGCatggtgagcaagggcgaggc	
	InFu_AP2g_mScarlet_R	GGATGATATTTAACGCGTcttgtacagctcgtccatgc	
	Ap2g_P2A_Nano_F	CAACAGGAATATTAGCGGCCGCGCCACAAACTTCTCTCTGCT	
	Ap2g_NanoLuc_R	GATGATATTTAACGCGTCGCCAGAATGCGTTCGCACAGCC	
Msrp1 (PF3D7_1335000)	MSRP1_12 gSeq_F	tattGAAATATTATATGAACAAAG	
	MSRP1_12 gSeq_R	aaacCTTTGTTCATATAATATTTC	
	MSRP1_HDR_F	GTACCGAGCTCgaattcGAACTTCTTACAAATAGCGA	
	MSRP1_HDR_R	TGCTCACCATacgcgtAAGTGTATTTAATAAATCCAC	
	MSRP1_3UTR_F	GCTGTACAAGaccggtTGAGATACATATGTATATATGTC	
	MSRP1_3UTR_R	CGAAAAGTGCCACCTgacgtcTGCATAATTCAATGAATATTCT	
	Msrp1_mNeonGreen_F	TAAATACACTTACGCGTATGGTGAGCAAGGGCGAGGAG	
	Msrp1_mNeonGreen_R	TATGTATCTCAACCGGTCTTGTACAGCTCGTCCATGCC	
**Genotyping**			
Ap2-g	*AP2-g Val_1 Endo F*	*ATGAGTTACAATAATCATTTTCG*	
	AP2-g Val Endo R	*TATAAATACATTTAGTTATAGGG*	
	TGP_Ap2g_Endo_R1	AATATCCATGTGTAAATAA	
	TGP_Ap2g_Endo_F1	AAATAATAATATCATTAACC	
Msrp1	CRI_MSRP1_Endo_F	GAACATCAGGATTACAAGGAG	
	CRI_MSRP1_Endo_R	CCCTACTTGAAGGAAAACCAGA	
Gdv1	GDV1_recoEndo_F	GATTTTGATTATGTGAGAG	
	GDV1_3UTR_Endo_R	AAATGGTATATACACCAAGG	
Stk2 or Stk2-KO	*P263 (P7)*	*GAAATCTATATAATGAGAAACATA3*	
	P264 (P8)	*ATTCTCTCTGTCTCCACCTTT3*	
	P261 (P5)	*CTTATACTTTTGAGTAGTTGT*	
	P262 (P6)	*TCATAGTTCACATTTCTTGTT*	
Plasmid's primer for 5'-integration test	HDR_R	CGAAAAGTGCCACCTGACGTC	
	P283	AGGGTTATTGTCTCATGAGCGG	**This work**
	B65_R	*GTAGACCCCATTGTGAGTAC (1)*	1

Gene expression			Ref
Ap2-g	Ap2-g_F	CCTGTATACCCTTCTTCGAAAGC (2)	
	Ap2-g_R	TCAAAAGTGCTCTCCTTTCGTG (2)	
Surfin 13.1	Surfin_13.1_F	ACCCGAAGTGACAACATCTCC (2)	
	Surfin_13.1_R	TCTCCACGAGTTCCAAGTTTT (2)	
Surfin 1.2	Surfin_1.2_F	TTTTTCCCTCGATCTCCGCG (2)	
	Surfin_1.2_R	GGGTTTGGCCGTACTCTACT (2)	
*Hda_*	HDA_F	AATAATGTGGGCGTGCAAGC (2)	
	HDA_R	TTCCTTGCATACCACCCGTT (2)	
Arom	AROM_F	CCAAAACGGGCGTAATGAACA (2)	
	AROM_R	GGGTGACTATTCCCATGATCGT (2)	
Ep2	EP2_F	ATGCGAGAAACATCCAGATGA (2)	
	EP2_R	TTACAATCCGACCTACAAAGACT (2)	
Pfge7	Pfge7_F	GGCCAAAATGAGGATGTTGA (2)	
	Pfge7_R	CGCTAGTATTGGGTGAAGCA (2)	
Gexp5	Gexp5_F	GTGGTTGTTTGAGAAGTGGTGA (2)	
	Gexp5_R	ACAGAATCCGTTTGAGATGATGA (2)	
Msrp1	MSRP1_F	TACCAGGTGCCTTATCAAGTG (2)	
	MSRP1_R	CTTGGTTGTGATTCGGTTGATG	
Sbp1	Sbp1_F	GGCATCTGCAACTACCGAAT (2)	
	Sbp1_R	GCTTGAAAAACCGTCATCGT	2
Arginyl-tRNA synthetase	PF3D7_1218600-Fw	AGCTAAAGAGATGCATGTTGGTCATT (1)	
	PF3D7_1218600-Rv	GAGTACCCCAATCACCTACATGA	1

(1) Usui M*, et al.* Plasmodium falciparum sexual differentiation in malaria patients is associated with host factors and GDV1-dependent genes. *Nat Commun* **10**, 2140 (2019).

(2) Prajapati SK*, et al.* The transcriptome of circulating sexually committed Plasmodium falciparum ring stage parasites forecasts malaria transmission potential. *Nat Commun* **11**, 6159 (2020).
